# The Place of Extensive Surgery in Locoregional Recurrence and Limited Metastatic Disease of Breast Cancer: Preliminary Results

**DOI:** 10.1155/2015/782654

**Published:** 2015-03-18

**Authors:** M. Berlière, F. P. Duhoux, L. Taburiaux, V. Lacroix, C. Galant, I. Leconte, L. Fellah, F. Lecouvet, D. Bouziane, Ph. Piette, B. Lengele

**Affiliations:** Breast Clinic, King Albert II Institute, Cliniques Universitaires Saint-Luc, 1200 Brussels, Belgium

## Abstract

The aims of this study were first to clearly define two different entities: locoregional recurrences and limited metastatic disease and secondly to evaluate the place of extensive surgery in these two types of recurrence. *Material and Methods*. Twenty-four patients were followed from June 2004 until May 2014. All patients underwent surgery but for 1 patient this surgery was stopped because the tumour was unresectable. *Results*. The median interval between surgery for the primary tumour and the locoregional recurrence or metastatic evolution was 129 months. Eight patients had pure nodal recurrences, 4 had nodal and muscular recurrences, 5 had muscular + skin recurrences, and 8 had metastatic evolution. Currently, all patients are still alive but 2 have liver metastases. Disease free survival was measured at 2 years and extrapolated at 5 years and was 92% at these two time points. No difference was observed for young or older women; limited metastatic evolution and locoregional recurrence exhibited the same disease free survival. *Conclusion*. Extensive surgery has a place in locoregional and limited metastatic breast cancer recurrences but this option must absolutely be integrated in the multidisciplinary strategy of therapeutic options and needs to be planned with a curative intent.

## 1. Introduction

Locoregional recurrences and limited metastatic disease represent a very complex challenge in the treatment of breast cancer. There is absolutely no consensus in the literature about the management of these entities, as there are many retrospective studies and no multicentric studies. Moreover, there is a big confusion between local recurrences, locoregional recurrences, new primary cancer, and limited metastatic disease. Recently, 24 experts from the Maastricht Breast Cancer Endpoint Consensus Group [1] defined local recurrences, second primary breast cancer, locoregional recurrences, and metastatic disease. According to the experts, local events are represented by events in the ipsilateral breast, the scar, and cutaneous nodes. These local recurrences are excluded from our study. Locoregional events concern ipsilateral nodes, axillary, infraclavicular, subclavicular, retropectoral, and internal mammary nodes (and also muscle recurrences in the pectoralis major and pectoralis minor). Distant recurrences, in fact metastatic evolution, concern invasion of contralateral nodes and of the sternal bone.

The aims of the study were to correctly and clearly define locoregional recurrences and limited metastatic disease. The second aim was to study the place of an extensive surgical approach in the therapeutic strategy and to evaluate its impact on disease free survival (DFS) and overall survival (OS).

## 2. Material and Methods

We performed our study on 24 consecutive patients followed from June 2004 to May 2014 for locoregional breast cancer or limited metastatic disease (chest wall) in our institution. The local ethics committee approved this study and the patients gave written informed consent to publish these data. The procedure to obtain a clinicaltrials.gov (http://clinicaltrials.gov/) identifier is currently ongoing.

After their primary breast tumour at this time, the patients were not yet included in our study; all patients were followed by clinical examination and blood tests every 3 months for 2 years, then every 6 months for 5 years and yearly thereafter, and yearly by breast mammogram and ultrasound. Breast MRI was added in case of suspicion of recurrence. After histological diagnosis of recurrence, the examination was completed by CT-scan of the chest and abdomen and bone scintigraphy. In case of metastatic evolution, PET-CT was also performed. In some cases, MRI of the bone marrow was also added. After treatment of the locoregional or metastatic recurrence, patients were followed every 3 months for 2 years and thereafter every 6 months according to the same modalities of follow-up (CT chest + abdomen, bone scan, blood tests, breast mammography + ultrasound, and systematically MRI).

*Statistical analysis* was performed using R Core Team. (R: A language and environment for statistical computing. R Foundation for Statistical Computing, Vienna, Austria, 2014 URL http://www.R-project.org/).

Kaplan-Meier curves were generated to calculate DFS.

## 3. Results

(a) Characteristics of the primary tumour and of the patients are described in Table 1. 24 patients were included in this study. A majority of them (62.5%) were menopausal. Sixteen out of the 26 initial surgical procedures were radical (2 bilateral mastectomies). Nine surgeries resulted in an axillary node-positive staging. All but one tumour had been endocrine receptor positive. Eighteen tumours benefited from adjuvant radiotherapy. Seven patients were treated with adjuvant chemotherapy and all patients were treated with adjuvant endocrine therapy.

(b) Characteristics of the recurrences (locoregional recurrences or metastatic evolution) are described in Table 2. At the time of their recurrence, only 2 patients were still premenopausal. The median interval between the initial occurrence of breast cancer and the recurrence was 129 months (range 24–286 months). Two recurrences were estrogen receptor negative.

**Figure 1:**
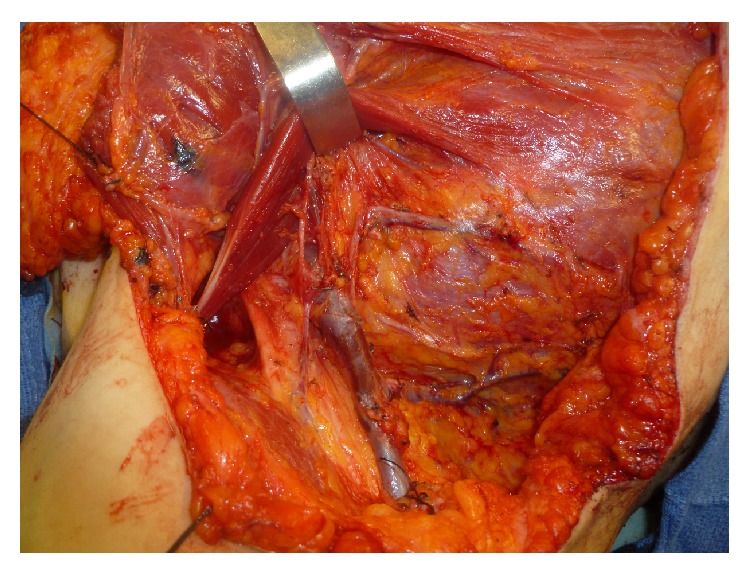
Subclavicular, retropectoral, and axillary dissection for axillary and subclavicular recurrence.

Of note, local recurrences were excluded from this study. Locoregional recurrence and metastatic evolution were defined according to the criteria of the Maastricht Group, Figure 1.

**Figure 2:**
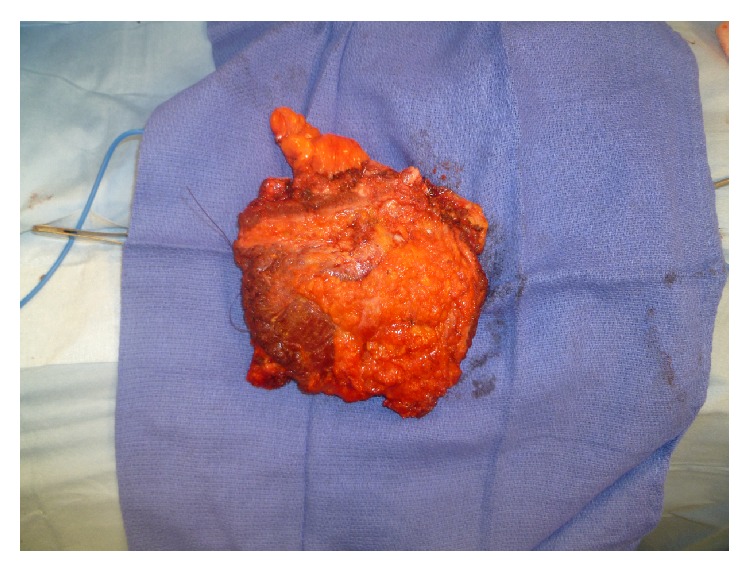
Resection of the anterior chest wall: inferior sternal bone and ribs (2, 3, 4, and 5) resection for large tumour involvement of the sternal area.

**Figure 3:**
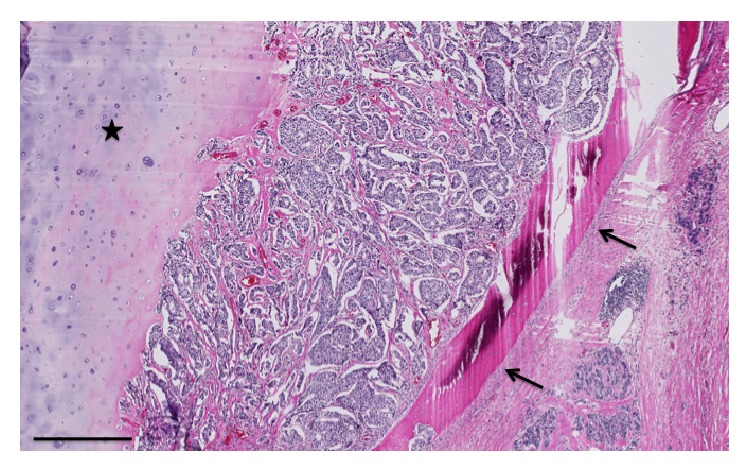
Low modification of carcinomatous infiltration of sternal bone as well as of the cartilage of the rib. The glandular neoplastic cells infiltrate and destroy the bone (indicated by arrows) and the cartilage (indicated by a star) (haematoxylin eosin staining, barr: 500 microns).

**Figure 4:**
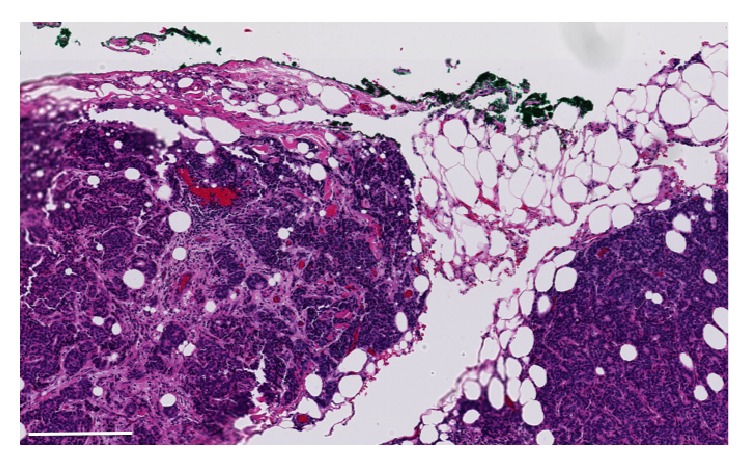
Histological view of pericardial infiltration, showing nests of neoplastic cells into the adipous tissue. The inked margin is free (Hematoxylin and Eosin staining, barr = 250 microns).

Eight patients had pure nodal recurrences, 4 had nodal + muscular recurrences (invasion of the pectoralis major), 5 had muscular and skin recurrences, and 8 had metastatic evolution (bone invasion, pleural effusion, etc.) Figures 2, 3 and 4.

(c) Staging of the disease is described in Table 2. All patients had breast MRI and CT-scan of the chest wall and of the abdomen. Twenty-two patients were additionally staged with PET-CT and 7 with MRI of the bone marrow.

(d) Morbidity of the surgical procedure was very low, as only 2 patients had a limited necrosis of latissimus dorsi flaps.

(e) Systemic therapies administered at the time of recurrence are mentioned in Table 3.

Systemic therapy was discussed during weekly multidisciplinary tumour board meetings. Surgery was thus integrated in a multimodality approach. A total of 20 patients received chemotherapy. Two options were discussed: either upfront surgery or initial chemotherapy. Fourteen out of the 24 patients received radiotherapy (50 Gy in case of mastectomy and 60 Gy in case of lumpectomy). For 6 patients, this actually was a second course of radiotherapy but overlap of the fields concerned only 1 patient who therefore received her second course of radiotherapy by brachytherapy.

For the 5 other patients who were administered a second course of radiotherapy, the first course of radiotherapy had only been administered on the breast and not on the nodal areas. There was thus no overlap of the fields. Twenty-two patients received endocrine therapy, which is still in progress for 20 of them. It was stopped for 2 patients with metastatic evolution (hepatic evolution). One of them received taxanes (6 courses) and thereafter, capecitabine, which is still in progress. The second patient with hepatic evolution is still receiving capecitabine.

(f)* Survival Rates*. With a median follow-up of 40.7 months (5–120 months), this study has a relatively short follow-up.

All patients were still alive at the time of database lock but 2 patients had a metastatic evolution with liver involvement. These liver metastases were treated by chemotherapy and the 2 patients currently have stable disease.

**Figure 5:**
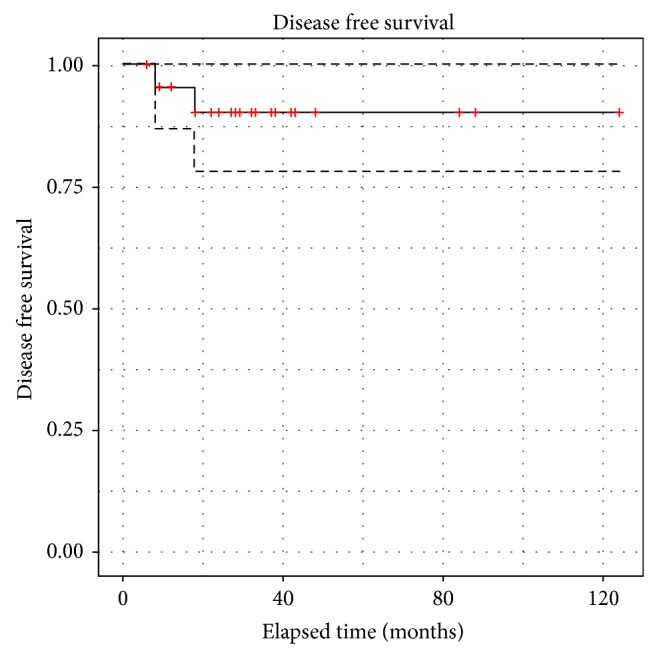
Disease free survival of all patients/locoregional recurrences and limited metastatic disease.

**Figure 6:**
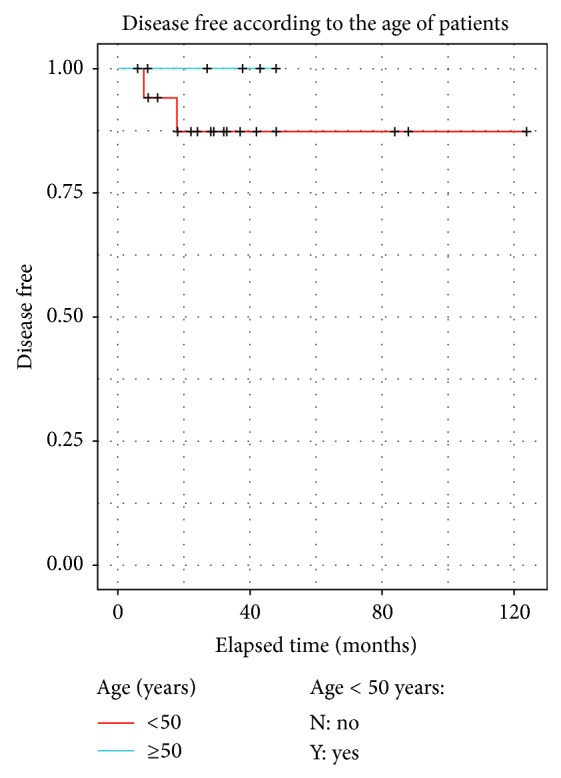
Disease free survival according to age (age <50 years and ≥50 years).

**Figure 7:**
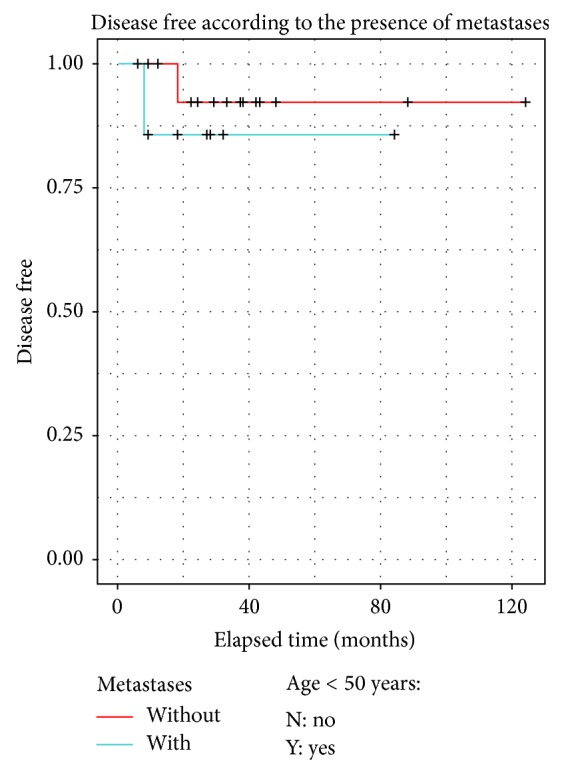
Comparison of the disease free survival between locoregional recurrence and limited metastatic disease.

DFS was calculated using Kaplan-Meier curves. No statistical difference was observed for young versus older patients and there was no difference between limited metastatic evolution and locoregional recurrences (Figures 5, 6 and 7).

## 4. Discussion

This study emphasizes the importance of a careful definition of locoregional recurrence and metastatic evolution (limited to the chest wall). Patients with local recurrences were excluded from this study.

The question of the exact incidence of this disease entity could unfortunately not be entirely resolved by this study, as some patients were referred by other cancer centres at the moment of their recurrence.

Locoregional recurrences can occur both after mastectomy and after breast-conserving surgery. In the past, the incidence was estimated between 5 and 40% [2]. Fortunately, the locoregional recurrence rate following breast cancer surgery has decreased with time [3]. This decrease is due to increased screening leading to the diagnosis of less advanced disease and to adjuvant therapies such as radiotherapy and also systemic therapies such as chemotherapy and endocrine therapy. This has been demonstrated in a retrospective study [3] that included 1,143 women younger than 40 years old with early breast cancer who underwent breast-conserving therapy in the South of the Netherlands between 1988 and 2010. After a median follow-up of 8.5 years, 186 patients had developed an isolated locoregional recurrence. The 5-year locoregional recurrence rate for the subgroup treated in the periods 1988–1998, 1999–2005, and 2006–2012 was, respectively, 9.8%, 5.9%, and 3.3%. In a multivariate analysis, adjuvant systemic therapy was associated with a reduction in the risk of locoregional recurrence of almost 60%. The locoregional recurrence rate is slightly lower after mastectomy than after breast-conserving therapy [4–6].

In the database of our breast cancer center (unpublished data), we observed the same decrease in locoregional recurrences over time. Between 2000 and 2005, the 5-year locoregional recurrence rate was 4% after breast-conserving surgery and mastectomy; it dropped to 2.5% between 2006 and 2011 [7]. These results are similar to those observed in the literature.

Careful staging of recurrences (locoregional and metastases of the chest wall) represents a crucial step in the management of these patients. In case of metastatic evolution, we cannot stress enough the importance of the preoperative management with CT-scan and PET-CT and of a multidisciplinary approach. Breast imaging (breast MRI, ultrasound) plays a major role in the identification and the localization of the recurrence.

Thanks to these careful screening procedures, we only had 2 undiagnosed pleural and pericardial effusions in our series (Figure 1); free margins were obtained for all patients except for these 2 patients. The results of our series, which is very small, are in general better than those published in the literature [7]. We therefore insist on the preoperative staging and the importance of the multidisciplinary approach. We think that resection of the chest wall with sternal bone and ribs (Figures 2 and 3) must be limited to patients treated with a curative intent and must not be considered as a palliative option [8–10].

The main objective of this extensive surgery is to achieve free margins in case of locoregional recurrences and in case of metastatic evolution. This highlights the importance of the surgical procedure. In all surgical procedures, the surgical resection is performed by a multidisciplinary team comprising a reconstructive plastic surgeon, a breast surgeon and, in case of thoracic invasion, a thoracic surgeon.

For nodal recurrences, we observed that the great majority of nodal recurrences are axillary and retropectoral. One case of subclavicular recurrence was also observed. In all cases, the three areas are explored in an attempt to obtain free margins (Figure 4). This was achieved in all cases. These results are similar to those reported in the literature.

The first therapeutic option, in case of isolated nodal recurrence, is to perform surgery [7, 11].

In case of muscular invasion, myocutaneous flaps are very often used to cover the defects and also to assure clear margins (aim achieved).

As described in the literature by Friedel, we think that the surgeons' expertise can improve survival and decrease morbidity [8, 9].

In our study, morbidity is very low because we only observed 2 limited necroses of latissimus dorsi flaps, treated by local care. No reintervention was necessary.

Follow-up of our study is short and the number of patients is small. Until now, we observe excellent results in terms of survival rate and disease free survival.

Our results are in agreement with the best results observed in the literature [8] and better than some studies, with a 5-year DFS at 92% (Figure 5). We think that careful preoperative staging and exact definition of locoregional recurrences and metastatic evolution are important because, in many manuscripts, there is a confusion between these 2 entities [8, 9].

This study is too small to clearly identify prognostic factors. As in Friedel's study, young age is not associated with bad prognosis (Figure 6).

If the patient has not previously received radiotherapy, radiation must be administered postoperatively, as already proposed by most authors. The irradiation involves the chest wall and the lymph nodes [12–15].

If the patient has undergone previous irradiation, the problem of the toxicity must be debated. In our institution, we do not repeat radiotherapy, except for brachytherapy. Only one patient had overlap of the fields. The other patients who had previously received radiotherapy were readministered radiotherapy but essentially on the axillary subclavicular and internal mammary nodes, which were not previously irradiated. It appears that irradiating with small doses could be recommended [13]. Photodynamic therapy is dedicated to skin cancer and its use in the context of locoregional recurrence is limited. Currently, only small series have studied this therapeutic option [16, 17].

In our series, limited metastatic invasion of the chest wall did not exhibit worse prognosis (Figure 7) than locoregional recurrences. The M1 status should thus not discourage the multidisciplinary team from proposing extensive surgery with a curative intent.

## 5. Conclusions

The results of our small study are very encouraging concerning disease free survival. This is the main information derived from this study. Our study needs to be completed by a prolonged follow-up and by an increased number of patients with a prospective follow-up. By the end of the year, 10 new patients will be added.

This study highlights the importance of the preoperative staging in an attempt to obtain free margins during the extensive surgery. The absolute necessity of a multidisciplinary team for the therapeutic decision and for the surgical procedures is also required to define the best therapeutic option for each patient.

## Figures and Tables

**Table 1:** Patients' and tumours' characteristics at initial breast surgery.

Age		
(i) <50	6	
(ii) ≥50	18	

Premenopausal status	9	
Menopausal status	15	

Histological subtypes (26 tumours analysed, 2 bilateral breast cancers)		Grade (i) Grade I: 2 (ii) Grade II: 18 (iii) Grade III: 6 Hormonal receptors (i) ER (+): 25 (ii) PR (+): 22 (iii) ER (−) + PR (−): 1 (DCIS grade III)
(i) Infiltrating ductal carcinoma	19	
(ii) Mixed lobular and ductal carcinoma	2	
(iii) Infiltrating lobular carcinoma	4	
(iv) Pure DCIS	1	
	

Initial breast surgery		
(i) Surgical procedures	26	
(ii) Breast-conserving surgery	10	
(iii) Mastectomy (2 bilateral)	16	

Axillary dissection performed in 26 cases		
(i) Negative nodes	17	
(ii) Positive nodes	9	

Quality of resection		
(i) R0	25	
(ii) R1 (1 mm DCIS)	1	

Adjuvant therapy		
(i) Radiotherapy		
(a) Yes	18	
(b) No	8	
(ii) Chemotherapy		
(a) Yes	7	
(b) No	17	

Endocrine therapy (24 patients)		
(i) Tamoxifen	22	
(ii) Tamoxifen + GnRH agonist	1	
(iii) Aromatase inhibitors	1	

R0: complete resection of any visible tumour with a pathologic demonstration of negative resection margins.

R1: complete resection of any visible tumour with a pathologic demonstration of positive margins.

ER: estrogen receptor.

PR: progesterone receptor.

**Table 2:** Patients' and tumours' characteristics at the time of recurrence.

Age		
(i) <50	2	
(ii) ≥50	22	

Premenopausal status	2	
Menopausal status	22	

Free interval between primary tumour and recurrence (months)		
(i) Median duration	129	
(ii) Range	24–242	

Histological subtypes (24 tumours analyzed)		Grade
(i) Infiltrating ductal carcinoma	18	(i) Grade I: 0
(ii) Infiltrating lobular carcinoma	4	(ii) Grade II: 7
(iii) Mixed	2	(iii) Grade III: 17

Endocrine status		
(i) ER (+)	22	
(ii) PR (+)	20	
(iii) ER and PR (−)	2	

Node dissection (24 patients)		
(i) Axillary retropectoral dissection	14	
(ii) Mediastinal intramammary chain dissection	7	
(iii) Node dissection not performed	3	
(iv) Bone resection (sternal and rips resection)		
(a) Yes	7	
(b) No	17	

Margin		
(i) R0	21	
(ii) R1	2	
(iii) Stop surgery for unresectable lesion	1	
(iv) Bone infiltration		
(a) Yes (bone and rips)	7	
(b) No	17	

Staging of the disease		
(i) Breast MRI	24	
(ii) Abdominal and chest wall CT-scan	24	
(iii) PET-CT	22/24	
(iv) MRI of bone narrow	7	

Morbidity of the surgery	2 limited necroses of flaps	

**Table 3:** Systemic treatment modalities at the time of recurrence.

External radiotherapy	
(i) Yes	13
(ii) No	10

Brachytherapy	1

Chemotherapy	
(i) First	4
(ii) After surgical procedure	16

Endocrine therapy (22 patients)	
(i) Aromatase inhibitors	22
	20/22 (still in progress)
	2/22 (stopped in patients with metastatic evolution)
